# The Impact of Moral Hazard on Healthcare Utilization in Public Hospitals from Romania: Evidence from Patient Behaviors and Insurance Systems

**DOI:** 10.3390/healthcare12242519

**Published:** 2024-12-12

**Authors:** Daniela Huțu, Carmen Marinela Cumpăt, Andreea Grădinaru, Bogdan Rusu

**Affiliations:** 1 Faculty of Economics and Business Administration, Alexandru Ioan Cuza University, Carol Boulevard, nr. 11, 700506 Iasi, Romania; daniela.diac@student.uaic.ro; 2 Department of Medical Specialties III, “Grigore T. Popa” University of Medicine and Pharmacy, 700115 Iasi, Romania; 3 Department of Quality Management, Clinical Rehabilitation Hospital, 700661 Iasi, Romania; gradinaru.andreea@scr.ro; 4 Faculty of Industrial Design and Business Management, “Gheorghe Asachi” Technical University of Iași, 700050 Iasi, Romania; bogdan.rusu@academic.tuiasi.ro

**Keywords:** moral hazard, healthcare, Oregon Medicaid Experiment, insurance, healthcare policy

## Abstract

Background/Objectives: Moral hazard represents a significant challenge in healthcare systems globally, reflecting the tendency of insured individuals to over-utilize medical services when shielded from the full costs of care. Methods: This paper investigates the dynamics and implications of moral hazard within the Romanian public hospital sector, offering practical recommendations for healthcare policymakers to mitigate the financial risks associated with excessive healthcare utilization and ensure long-term sustainability. To achieve the objectives of this study, a quantitative research approach utilizing vignettes was employed. Vignettes allow for the simulation of real-world decision-making under conditions of insurance coverage, capturing nuanced behaviors that traditional surveys may overlook. Results: The study examined patient behaviors in the context of moral hazard in public hospitals in Romania, employing a quantitative approach based on vignettes. A total of 303 valid responses were collected. The findings indicate a significant tendency among insured patients, both publicly and privately insured, to opt for more expensive treatments compared to uninsured patients, who preferred more affordable options such as medication or physiotherapy. In the case of treatments for severe conditions, insured patients frequently chose combinations of higher-cost therapies, while uninsured individuals either delayed treatment or opted for less expensive alternatives. These results highlight the impact of moral hazard, driven by a reduced sensitivity to costs in the presence of insurance, and underscore the need for cost-sharing policies to mitigate the overutilization of medical resources. Conclusions: This paper uniquely contributes to the understanding of moral hazard by integrating insights from both Romanian public hospitals and international case studies, offering practical policy recommendations for mitigating the financial risks associated with excessive healthcare utilization.

## 1. Introduction

Moral hazard in healthcare refers to the tendency of individuals to alter their behavior due to the protection provided by health insurance, potentially leading to inefficient utilization of medical resources. Insured individuals become less concerned about the costs of care, thereby fostering overuse of services and neglect of preventive measures. This concept, introduced by Kenneth Arrow, highlights the increased demand for healthcare services as a result of insurance coverage [1,2]. In this context, moral hazard manifests in costly and sometimes unnecessary treatments, exacerbating healthcare system expenditures [3].

While moral hazard has been extensively studied in developed countries, limited research has addressed its manifestation in transitioning economies such as Romania, where public insurance plays a central role. This study analyzes the manifestations of moral hazard within the specific context of Romanian public hospitals and proposes strategies to mitigate its impact on service utilization and system sustainability. In contrast to the well-funded healthcare systems of many Western European countries, the Romanian public healthcare system faces significant challenges due to chronic underfunding and systemic inefficiencies, rendering it particularly vulnerable to moral hazard. Operating under a social health insurance model financed through employer and employee contributions, Romania’s healthcare system remains under-resourced compared to other member states of the European Union. Public hospitals contend with inefficiencies that result in elevated healthcare costs and disparities in access to essential services. These structural vulnerabilities create conditions conducive to moral hazard, wherein both patients and healthcare providers may engage in practices that exploit insurance coverage, exacerbating service overutilization and further straining the already burdened system. International studies suggest that reducing out-of-pocket costs through insurance results in a significant increase in the consumption of healthcare services, including non-essential ones [4,5].

Einav and Finkelstein suggest that the term “moral hazard” is widely used to describe the notion that insurance coverage, by reducing the marginal cost of care for the individual (often referred to as the out-of-pocket cost of care), can lead to increased healthcare utilization. Health insurance may also incentivize individuals to exert less effort in maintaining their health. This shift in economic incentives not only promotes greater consumption of healthcare services but also discourages personal responsibility in maintaining health [1]. For instance, health insurance may diminish the perceived financial consequences of unhealthy behaviors, such as smoking or a sedentary lifestyle, thereby reducing individual motivation to adopt preventive health measures [5].

In addition to patients, health insurance also influences healthcare providers. Cutler and Zeckhauser [6] argue that providers may choose more expensive treatments or diagnostic tests, knowing that insurance will cover the costs, a phenomenon known as “supplier-induced demand” [3]. This behavior increases system costs and negatively impacts the sustainability of insurance schemes, particularly for low-income individuals [7].

The dual influence of moral hazard on both patients and providers highlights its critical role in the sustainability of healthcare systems. By mitigating financial risks, insurance can unintentionally promote riskier behaviors and heightened demand for services [8]. Addressing and managing this issue is essential for designing equitable and sustainable healthcare systems that ensure access without undermining financial viability.

Moral hazard arises when insured individuals can influence either the likelihood of an insured event occurring or the magnitude of the resulting financial loss. It manifests in two distinct forms: ex-ante moral hazard and ex-post moral hazard [9].

Ex-ante moral hazard refers to the tendency of insured individuals to adopt riskier behaviors, given that insurance reduces the financial burden associated with adverse health outcomes [3]. Behaviors such as unhealthy eating, physical inactivity, or smoking become more prevalent because individuals perceive that potential medical expenses will be covered by insurance. This phenomenon is particularly pronounced in systems offering comprehensive coverage with minimal cost-sharing by patients [10]. Studies indicate that insured individuals exhibit higher hospitalization rates for preventable conditions, reflecting reduced incentives to maintain a healthy lifestyle [11].

However, the actual impact of ex-ante moral hazard on individual behavior remains a topic of debate. Nyman argues that, while some individuals may take greater health risks, many are deterred by the non-financial costs of illness, such as income loss, physical suffering, and reduced quality of life [3]. For instance, the decision to smoke despite the risk of lung cancer cannot be entirely attributed to the mere existence of insurance, as the health consequences remain a significant deterrent [12].

In Romania, ex-ante moral hazard manifests as reduced engagement in preventive measures, such as regular medical check-ups or the adoption of a healthy lifestyle. Patients tend to prioritize reactive treatments, knowing that these are covered by insurance. This tendency reflects a broader pattern of neglecting prevention, a characteristic of transitioning economies.

The specialized literature offers limited empirical studies on ex-ante moral hazard, partly due to the challenges of quantifying the economic consequences of risky behaviors. This underscores the complexity of the relationship between the financial incentives provided by insurance, individuals’ behavioral choices, and the broader psychosocial costs of illness [1]. Addressing this phenomenon requires a deeper understanding of how financial incentives influence health behaviors and amplify inefficiencies in the utilization of medical resources.

Ex-post moral hazard arises after the onset of an illness or injury, when insured patients become less attentive to the costs of treatment decisions, relying on insurance to cover most expenses [1]. This form of moral hazard encourages more expensive treatment options, such as newer medications, over equally effective generic equivalents. Combined with ex-ante moral hazard, this behavior significantly contributes to rising healthcare costs and the overburdening of public resources [13].

In Romania, ex-post moral hazard is evident in the increased demand for diagnostic procedures and costly tests once patients are covered by public insurance, despite the availability of less expensive alternatives. Unlike ex-ante moral hazard, which influences behavior prior to the onset of illness, ex-post moral hazard is driven by needs that arise after the condition has manifested [6]. This behavior leads to the overutilization of medical resources, resulting in higher healthcare system expenditures and increased insurance premiums, which undermine the sustainability of public insurance schemes.

Research by Einav and Finkelstein highlights that the financial impact of ex-post moral hazard is significantly greater than that of ex-ante moral hazard. Patients often utilize insurance coverage to access more expensive treatments, even when the additional benefits are marginal. For instance, patients may opt for minimally invasive surgeries, which are costlier, over traditional procedures that are equally effective, perceiving reduced financial risk [1]. This tendency is also observed in Romania, where patients favor innovative treatments despite their higher costs, reassured by the certainty that insurance will cover these expenses.

While both forms of moral hazard contribute to inefficiencies in healthcare systems, ex-post moral hazard has an immediate and substantial impact on costs. Addressing these challenges requires integrated strategies, including cost-sharing mechanisms, patient education, and aligning incentives between healthcare providers and patients to promote efficient resource use and ensure long-term sustainability.

This paper provides a novel perspective on moral hazard in Romania by examining the impact of public and private insurance on patients’ medical decisions within public hospitals. Unlike general studies on moral hazard in developed economies, this research addresses a relatively unexplored topic in transitioning economies. A significant contribution of this study is the use of a vignette-based methodology, which enables the simulation of medical decisions across different insurance scenarios. This approach provides a detailed understanding of how insurance status influences treatment choices, thereby contributing to the development of policies aimed at enhancing the sustainability of Romania’s healthcare system.

## 2. Healthcare Insurance Experiments

### 2.1. The Oregon Experiment

Over the years, various experiments have been conducted to investigate the effects of moral hazard within healthcare systems. One of the most renowned is the Oregon Health Insurance Experiment [1]. This experiment, conducted in 2008, provided a unique opportunity to study the impact of health insurance on healthcare utilization, particularly among low-income individuals. Oregon’s Medicaid program used a lottery system to randomly select uninsured citizens for Medicaid coverage, ensuring that selection was independent of participants’ health status [14]. Out of 74,922 individuals who applied, 29,834 were randomly selected to receive Medicaid benefits, allowing researchers to observe a naturally occurring randomized controlled trial in healthcare coverage [15].

The Oregon Health Insurance Experiment yielded several notable findings over the two-year observation period. While Medicaid coverage did not result in significant improvements in physical health outcomes—such as control of hypertension, cholesterol, or diabetes—it led to increased healthcare utilization and other key benefits. Participants experienced higher rates of diabetes detection and management, reduced rates of depression, and alleviated financial strain associated with healthcare costs [15]. However, a prominent result was the 40% increase in emergency department visits, even for conditions that could have been treated in outpatient settings or prevented altogether [16]. This increase in emergency department usage is often cited as evidence of moral hazard, illustrating how individuals, once insured, may overutilize certain healthcare services regardless of the necessity for those services.

The Oregon Experiment has been instrumental in illustrating the complexity of moral hazard within the healthcare sector. It highlights the trade-offs between providing essential financial protection for vulnerable populations and the unintended consequences of increased healthcare consumption, particularly in emergency settings.

### 2.2. The RAND Health Insurance Experiment

Preceding the Oregon experiment, the RAND Health Insurance Experiment (HIE) remains one of the most comprehensive studies to date on the relationship between insurance structure and healthcare utilization. Conducted from 1974 to 1982, the RAND experiment was designed to rigorously assess how different cost-sharing structures in health insurance affect the demand for healthcare services [17]. Unlike the Oregon Experiment, which focused on the expansion of Medicaid, the RAND study evaluated a range of cost-sharing plans, providing a broader view of consumer behavior under varying financial incentives.

In the RAND experiment, over 5800 participants from six U.S. regions were randomly assigned to insurance plans with differing levels of cost-sharing, ranging from full coverage (with no out-of-pocket costs) to plans requiring substantial co-payments [18]. The study demonstrated a clear inverse relationship between the level of cost-sharing and the utilization of healthcare services: participants with full insurance coverage (zero cost-sharing) utilized significantly more healthcare services than those in high cost-sharing plans. The elasticity of healthcare demand in response to out-of-pocket costs was evident, as individuals with higher cost-sharing obligations reduced their use of both necessary and unnecessary medical services [17].

However, the RAND experiment also emphasized that factors beyond direct medical costs, such as transportation and time spent accessing care, play a crucial role in determining healthcare utilization [18]. These additional costs—often overlooked in discussions of moral hazard—affect healthcare decisions as much as, if not more than, financial cost-sharing.

The RAND Health Insurance Experiment conclusively demonstrated the existence of moral hazard in healthcare: as consumer cost-sharing decreases, healthcare utilization and overall spending increase [1]. This moral hazard effect becomes particularly pronounced in the context of comprehensive insurance plans, where patients are more likely to consume healthcare services that they might otherwise forgo if they had to bear the full costs themselves. Notably, the experiment also showed that while cost-sharing reduced the use of medical services, it did not lead to significant negative health outcomes for most participants, challenging assumptions that higher utilization necessarily correlates with better health outcomes [17].

In summary, both the Oregon and RAND experiments provide critical insights into the complex relationship between insurance coverage, healthcare utilization, and moral hazard. While increased coverage improves access to care and alleviates financial stress, it also promotes the overuse of services, underscoring the need for carefully balanced policy interventions that account for both the benefits and risks associated with comprehensive health insurance.

## 3. Romanian Healthcare System

The Romanian healthcare system operates on a social health insurance model, primarily funded through mandatory contributions made by both employers and employees [19]. In 1997, Romania adopted the Bismarck model, a system rooted in compulsory health insurance and the principle of solidarity, ensuring access to healthcare services through a decentralized structure. This model is prevalent across several European Union countries, including Belgium, Germany, France, the Czech Republic, Estonia, Lithuania, Luxembourg, Poland, the Netherlands, Slovakia, Hungary, and Slovenia [20]. The adoption of this model marked a significant shift in Romania’s healthcare financing, aiming to enhance the efficiency and equity of healthcare delivery.

The primary challenge for the Romanian healthcare system, as for many other healthcare systems, is cost management. Healthcare costs in Romania are influenced by numerous factors; however, the present study focuses exclusively on the impact of health insurance within public hospitals in Romania [21]. The rising costs in Romania’s healthcare system are driven by a multitude of factors, including demographic changes, increasing demand for services, and inefficiencies in resource allocation. This study, however, narrows its focus to explore the role of health insurance in shaping healthcare utilization within Romanian public hospitals, where moral hazard plays a critical role [22].

Theoretically, insurance reduces the monetary cost individuals pay for healthcare services. However, this reduction may also contribute to “moral hazard,” whereby individuals engage in riskier behaviors due to the shared or absorbed nature of the financial risk [1]. The phenomenon of moral hazard is particularly relevant in public healthcare systems like Romania’s, where both patients and providers may alter their behaviors due to the presence of insurance coverage.

Insurance beneficiaries may be tempted to utilize healthcare services more frequently or request costly procedures and treatments excessively, often without medical justification. Similarly, healthcare providers or physicians may prescribe unnecessary or excessive tests, procedures, or treatments, knowing that the costs will be covered by insurance, thereby generating higher profits [23].

Such behavior can lead to increased healthcare system costs, as excessive use of medical services may become financially unsustainable and could limit access to essential services for other individuals [24,25].

The objective of this study is to assess whether the effect of health insurance on health-related behaviors in Romania aligns with findings reported in the existing literature. In other words, the study aims to explore a relatively under-researched area: moral hazard and the behaviors exhibited by individuals in Romanian public hospitals regarding the consumption of healthcare services.

## 4. Materials and Methods

To achieve the objectives of this study, a quantitative research approach utilizing vignettes was employed. Vignettes allow for the simulation of real-world decision-making under conditions of insurance coverage, capturing nuanced behaviors that traditional surveys may overlook [26]. As Finch (1987) defines them, vignettes are short, descriptive stories that outline hypothetical situations under specific conditions to which participants are invited to respond. This approach proves particularly useful in exploring sensitive topics that participants might otherwise find difficult to discuss openly [27].

Vignette methodologies have been increasingly adopted in healthcare research to assess decision-making under various scenarios, offering a robust way to capture nuanced patient behaviors that might not be evident through traditional survey methods [28]. Vignettes are also recognized as an effective method for assessing clinical decision-making, particularly in healthcare contexts, where they provide insight into complex professional judgments [28,29].

**Figure 1:**
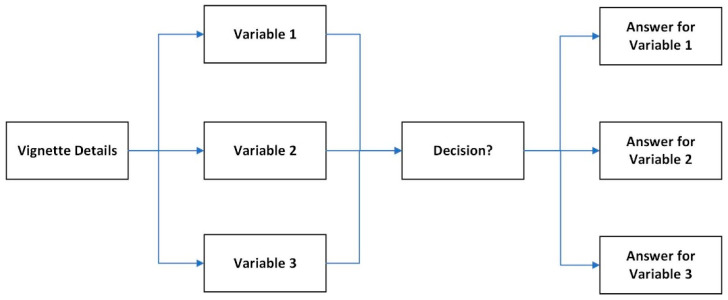
Vignette structure with three variables (adapted from [31]).

Vignettes have been widely employed to evaluate the quality of care both within and across countries, enabling comparative analyses of healthcare standards on an international scale [30]. Their ability to mimic realistic clinical scenarios makes them a valid and powerful tool for assessing healthcare outcomes and behaviors. In the present study, vignettes were constructed to depict common medical conditions in Romania, wherein potential patients were required to make decisions while considering three different insurance statuses: public health insurance, private health insurance, or no insurance at all. Figure 1 illustrates the structure of the vignette, including the three variables used.

To capitalize on the strengths of both experimental and traditional research methods while minimizing their limitations, they were integrated into a questionnaire-based survey (see Supplementary Materials). This hybrid approach allowed for the collection of nuanced data on healthcare decisions while ensuring broad accessibility and ease of response.

The questionnaire comprised three distinct sections. In the first section, participants who were employees of the public healthcare system were provided with an informed consent form detailing the study’s procedures, the time commitment required, the risks and benefits of participation, the nature of the information requested, and their right to withdraw from the study at any time. These participants were selected because of their familiarity with the medical terminology presented in the vignettes. Participants were also assured that their responses would be confidential, their anonymity preserved, and that all personal data would be handled in compliance with European data protection regulations (GDPR). Those who responded to the survey verbally consented to participate, acknowledging their understanding of the study’s terms and conditions. Following a review of the responses provided, no respondents were identified as failing to complete the vignettes; thus, no exclusions from the sample were necessary.

The second section focused on collecting socio-demographic information. Participants were required to respond to six closed-ended questions with predefined response options to ensure the consistency and comparability of data.

In the third section, respondents were presented with three distinct vignettes, each depicting a medical situation in narrative form. The vignettes were developed by the authors, drawing upon both the studies available in the specialized literature and their personal experience, with three of the four authors actively working in the Romanian public healthcare system. These scenarios were carefully designed to simulate real-world healthcare decision-making processes and were constructed based on both relevant literature and clinical expertise, particularly with respect to issues such as autonomy, responsibility, and clinical outcomes [32]. The validity of vignette-based surveys depends heavily on the quality of the vignette design, including clarity, structure, and the methods used to present the vignette questions [33].

The vignettes included key components such as the patient’s medical history, the management of the condition up to the present, diagnostic test results (where applicable), and the costs associated with various treatment options. These components ensure that respondents can make informed decisions in the hypothetical scenarios. The selection of pathologies was informed by their high prevalence among patients in contemporary healthcare settings. Osteoarticular conditions, which are increasingly common, represent a significant clinical concern due to their painful nature and the necessity for timely intervention to prevent progression to advanced stages. Similarly, ENT (ear, nose, and throat) pathologies affect individuals across all age groups, ranging from neonates to older adults, and can substantially diminish the quality of life if appropriate medical measures are not implemented promptly. The vignette on renal tumors was designed to place respondents in a scenario where the disease threatens both quality of life and survival. While renal tumors are not among the most common cancers in Romania, they are recognized as particularly difficult to treat, emphasizing their clinical significance. Also, urological conditions are frequent in both men and women. Table 1 provides a summary of the vignettes used in the study.

The questionnaires were distributed electronically through the WhatsApp platform in August and September 2023, following the approval of the hospital’s Ethics Committee, which was granted in May 2023. Potential participants received the investigator’s contact information (phone and email) to facilitate communication and confirm their participation in the study. For non-responders, the questionnaire was re-sent after one week to encourage participation. The decision to utilize WhatsApp for electronic delivery was motivated by the convenience and broad accessibility of this platform, especially for reaching participants in remote areas who might otherwise be difficult to contact. In addition, electronic data collection offers the advantage of minimizing errors during database construction and enhances data accuracy through automated processes [34]. Following a review of the responses provided, no respondents were identified as failing to complete the vignettes; thus, no exclusions from the sample were necessary.

## 5. Results

Following the administration of the questionnaires, 303 valid responses were obtained for analysis.

**Figure 2:**
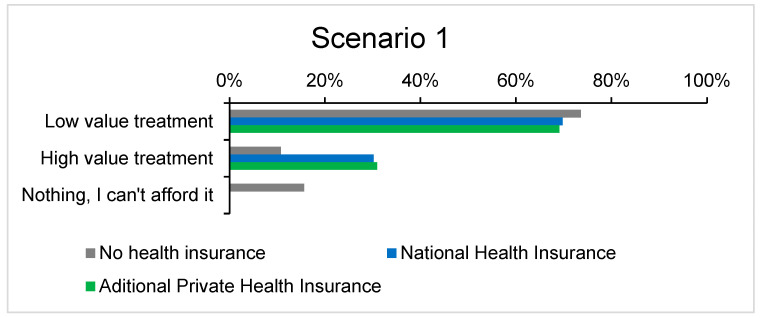
Selection of affordable vs. expensive treatment based on insurance type in Scenario 1.

Scenario 1 explored treatment options for a herniated disc, providing participants with a choice between less expensive treatments (such as physiotherapy or medication) and a more costly surgical intervention. The results reveal significant variations based on the participants’ insurance status. Most respondents who opted for the more expensive surgical treatment were those covered by public health insurance, followed closely by those with private insurance. Figure 2 highlights the difference in treatment choices between insured and uninsured patients, illustrating the potential presence of moral hazard among insured individuals. Insured participants were more likely to select surgical intervention as an immediate solution, knowing they would not bear the financial burden. Conversely, uninsured participants were least likely to choose surgery, opting instead for more affordable alternatives such as medication and physiotherapy. This behavior suggests the presence of moral hazard to some extent, as insured individuals, aware that their health insurance would cover the costs, tended to choose the safer, faster, but more expensive treatment option.

**Figure 3:**
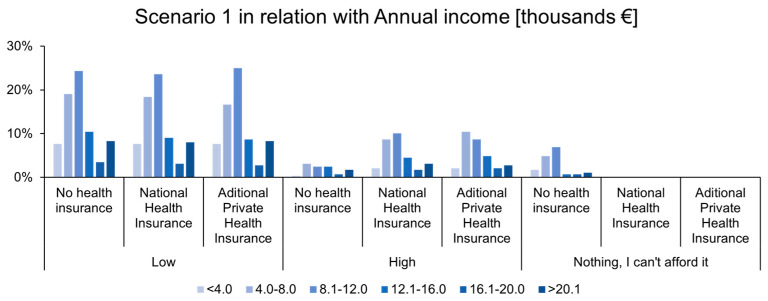
Results by annual income [thousands €].

On the other hand, a significant proportion of respondents—irrespective of insurance status (uninsured, publicly insured, or privately insured)—chose the combination of medication and physiotherapy. This decision does not necessarily imply the absence of moral hazard; rather, it may be influenced by personal factors such as fear of surgery, mistrust in the effectiveness of invasive treatments, or financial constraints, especially for uninsured individuals as shown in Figure 3. Furthermore, it may reflect a general preference for less invasive treatment options, even among insured individuals, who might choose to exhaust non-surgical avenues first. In this scenario, respondents’ choices may have been influenced not only by cost but also by a range of other factors, which can vary between individuals depending on the status of their condition. It is important to emphasize that any choice not medically justified contributes to the manifestation of moral hazard.

Interestingly, 6% of respondents indicated that they would take steps to obtain public health insurance to avoid paying out-of-pocket for healthcare services. This indicates that uninsured individuals recognize the financial barriers to treatment and may view insurance as essential for accessing adequate care.

The proportion of respondents who expressed interest in seeking a second medical opinion was notably low, at only 2%. However, none of these individuals were uninsured, suggesting that patients with insurance are more likely to pursue additional consultations as they know that their insurance will cover the cost. The low rate of uninsured patients seeking second opinions suggests limited accessibility and perceived futility in additional consultations, reflecting deeper healthcare equity challenges. In contrast, uninsured patients were less inclined to consider this option, likely due to the increased financial burden of out-of-pocket payments for a second opinion.

These findings imply that uninsured individuals may not fully explore all available medical options, potentially leading to suboptimal health outcomes. Addressing financial barriers could improve equity in healthcare utilization and ensure that all patients can make informed decisions about their care.

**Figure 4:**
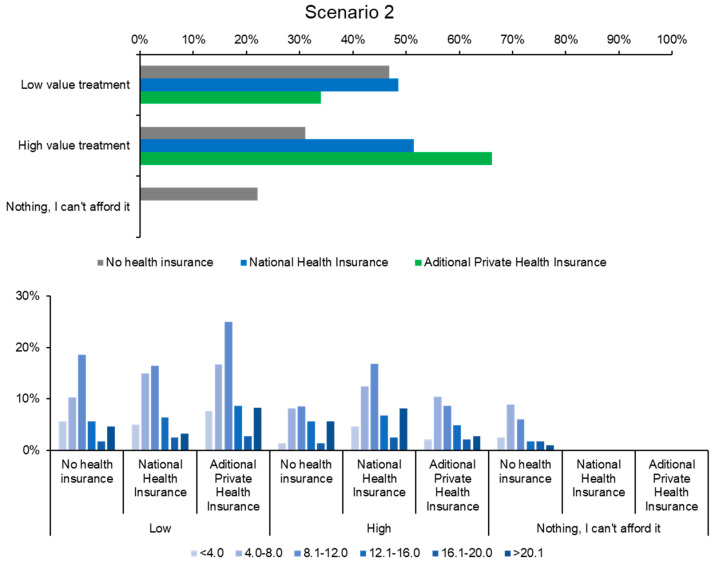
Selection of affordable vs. expensive treatment based on insurance type in Scenario 2.

Scenario 2 presented a hypothetical case involving the treatment of renal tumors following an MRI diagnosis. Respondents had to choose between standard chemotherapy (a less expensive option) and a more expensive treatment that involved combining chemotherapy with a new medication costing 15,000 RON per month. Figure 4 illustrates how uninsured patients predominantly opted for the less expensive chemotherapy, while insured patients preferred the more expensive combination therapy, highlighting the influence of insurance on decision-making and moral hazard. Some uninsured respondents explicitly stated that they chose standard chemotherapy due to financial constraints, while insured individuals selected the full treatment, confident that they would not need to bear the costs themselves.

Given the severity of the diagnosis, a small percentage of respondents chose to delay treatment. Only 1% of privately insured and 2% of publicly insured participants indicated that they would postpone treatment, primarily to seek additional medical opinions. In contrast, 7% of uninsured respondents delayed treatment, citing financial limitations as the main reason for postponement.

**Figure 5:**
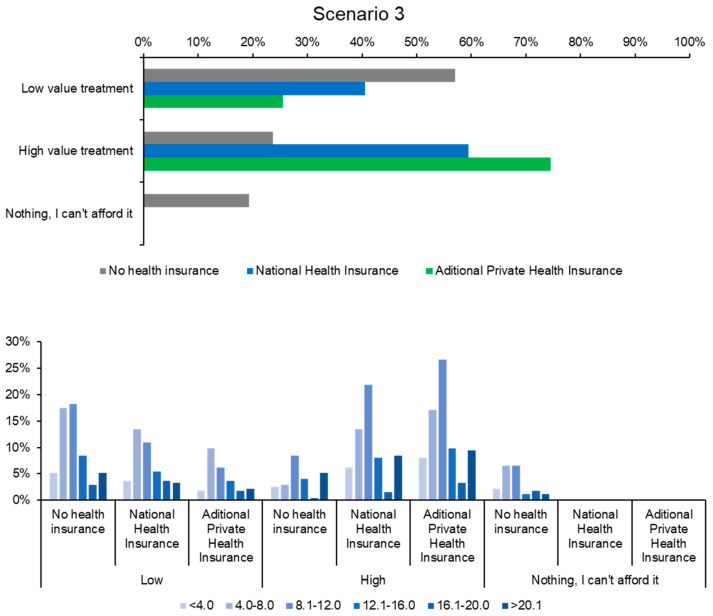
Selection of affordable vs. expensive treatment based on insurance type in Scenario 3.

This higher delay among uninsured patients may lead to worse health outcomes, as timely treatment is critical in life-threatening conditions like cancer as shown in Figure 5. The implication is that lack of insurance not only affects treatment choices but also impacts the timing of care, potentially exacerbating health disparities.

The findings from this scenario highlight the manifestation of moral hazard among insured individuals, who tended to opt for combination therapy, which may or may not have been medically justified depending on the case. In other words, insured respondents readily chose the more complex and expensive treatment without significant hesitation, relying on varying degrees of information or understanding regarding potential risks or adverse effects.

Of the total 303 respondents, 10% of uninsured participants indicated they would seek to obtain insurance to access treatment at a reduced cost compared to the expense of paying for care out of pocket. This suggests that severe health conditions can motivate uninsured individuals to obtain insurance, highlighting the role of insurance in facilitating access to necessary but expensive treatments.

When it came to seeking a second medical opinion, the percentage remained low, at only 1% across all categories (publicly insured, privately insured, and uninsured). This low rate may reflect a lack of awareness or trust in the healthcare system or cultural factors affecting patient autonomy and decision-making. Enhancing patient education about the benefits of second opinions could improve healthcare outcomes.

Given the life-threatening nature of Scenario 2, 22% of respondents stated that they would pursue any means necessary to afford treatment, including taking out loans or selling personal assets such as cars or houses. This figure is significantly higher compared to Scenario 1, where only 6% of respondents indicated they would seek additional financial resources. The willingness to incur significant financial burdens underscores the critical value placed on life-saving treatments and highlights the risk of catastrophic health expenditures for those without sufficient insurance.

Scenario 3 focused on hearing loss, with respondents asked to choose between a partially implantable Baha prosthesis (costing between 25,000 and 40,000 RON) and a conventional hearing aid (costing approximately 10,000 RON). Analysis of the responses revealed that uninsured participants overwhelmingly opted for the less expensive conventional hearing aid. In contrast, the more costly Baha prosthesis was the majority choice among those with public or private insurance, as shown in Figure 5. This scenario underscores the manifestation of moral hazard, as insured respondents assumed that the more expensive option was the best choice for regaining their hearing despite being unaware of whether this decision would provide superior clinical benefits compared to the alternative presented.

Although some insured respondents also chose the less expensive hearing aid, their decision was influenced by the fact that only the hearing aid would be reimbursed by insurance, while opting for the Baha prosthesis would entail additional out-of-pocket costs. Uninsured respondents, meanwhile, were largely driven by financial constraints, opting for the more affordable option despite its limitations as shown in Figure 5 This indicates that even among insured patients, the extent of coverage significantly affects treatment decisions, suggesting that partial coverage may not fully mitigate the impact of cost on healthcare choices.

Regarding treatment delays in Scenario 3, 13% of respondents indicated postponing the treatment. Among this group, 8% were uninsured individuals who explained that they would have to pay for hearing restoration out of pocket, which they either could not afford or deemed too expensive. Delaying treatment for non-life-threatening conditions like hearing loss can affect quality of life and social participation, showing how financial barriers can defer care and increase long-term healthcare costs. This highlights a common theme: uninsured individuals, in an attempt to access medical care without bearing the costs directly, often seek to obtain insurance coverage, underscoring the presence of moral hazard.

**Figure 6:**
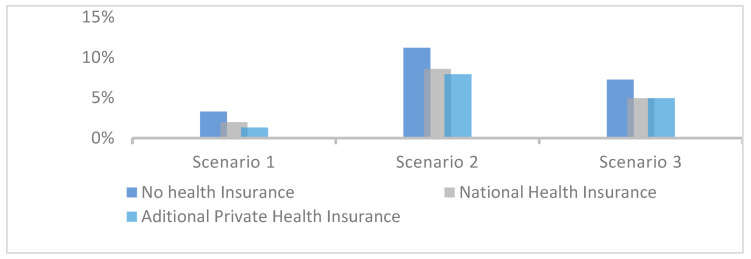
Percentage of patients resorting to alternative financial sources for treatment.

The percentage of respondents who would resort to alternative financial resources to fund treatment was 6%, lower than in Scenario 2 (9%) but higher than in Scenario 1 (3%) as shown in Figure 6. This indicates that respondents’ decisions are also shaped by psychological and behavioral factors, with varying degrees of risk tolerance depending on the severity of the situation. In life-threatening cases such as Scenario 2, a larger proportion of uninsured patients (9%) stated that they would pursue any means necessary to secure additional income for treatment. This was not the case in Scenarios 1 and 3, where the conditions posed no immediate threat to life, leading many uninsured patients to delay or forgo treatment. These findings emphasize the critical role that perceived severity of illness plays in healthcare decision-making and the potential for financial barriers to result in delayed or forgone care for less acute conditions. This observation suggests that moral hazard arises when insured individuals select more expensive treatments. However, such decisions do not constitute moral hazard if the chosen treatment demonstrates clinical superiority or offers distinct therapeutic advantages over the alternative.

## 6. Discussion

This study has addressed several key questions regarding the influence of health insurance on medical behaviors and healthcare consumption in public hospitals in Romania. The first question explored whether the effect of insurance on health-related behaviors in Romania aligns with findings reported in the specialized literature. The empirical results indicate that individuals in Romanian public hospitals make medical decisions primarily based on their annual personal income and the direct costs associated with treatment.

The data revealed that individuals with higher annual incomes were more likely to choose more expensive treatment options, regardless of their insurance type. Conversely, individuals with lower annual incomes opted for costlier treatments only if they were covered by public or private insurance. When treatments involved out-of-pocket expenses that exceeded insurance coverage, patients either postponed their decision or selected treatments that were fully reimbursed by their insurance, indicating a financial constraint in decision-making.

Moreover, when health insurance covered both the basic treatment and supplementary options, the majority of insured individuals preferred the more comprehensive and safer treatment, disregarding the associated costs. This behavior underscores the moral hazard phenomenon, where individuals consume more expensive healthcare services, knowing that the financial risks are mitigated by insurance.

The study also answered the second key question: insured individuals in Romanian public hospitals tend to use more medical services, sometimes to an excessive degree, and are more inclined to opt for costlier treatment options. This is a clear indication of moral hazard in the Romanian healthcare system, where patients overutilize services and make potentially costly and risky healthcare decisions, driven by the knowledge that their insurance will absorb a significant portion of the costs.

Excessive medical service consumption contributes to rising healthcare system costs, limits access to essential services for other patients and poses long-term financial challenges for the sustainability of public healthcare. These behaviors not only strain healthcare resources but also raise broader concerns about healthcare equity and access for uninsured or underinsured populations.

This study confirms that moral hazard significantly shapes health-related behaviors in Romanian public hospitals, reflecting trends seen in broader healthcare systems. A complex interplay of social, economic, and psychological factors, including income levels, insurance status, and perceptions of financial risk, drives the behaviors observed.

Furthermore, developing a tiered insurance model that distinguishes between essential medical care and elective procedures could lead to more judicious use of healthcare services. Requiring greater financial participation for non-essential treatments would help patients make more informed and prudent choices. Enhancing patient education about treatment decisions’ financial and health-related implications is also crucial. Empowering patients with transparent information on costs and outcomes can mitigate moral hazard effects by promoting more thoughtful decision-making.

However, this study’s scope has been limited to identifying the presence of moral hazard and assessing the impact of health insurance on healthcare utilization. Further research should explore regional disparities in healthcare consumption within Romania and potential cultural factors influencing patients’ healthcare decision-making and moral hazard behaviors. Addressing these variables in future studies would provide a more comprehensive understanding of how to mitigate moral hazard and promote more efficient and equitable healthcare delivery.

## 7. Conclusions

To address these challenges, policymakers in Romania should consider several key actions. First, introducing targeted cost-sharing measures, such as co-payments or deductibles for elective treatments, could help reduce the overutilization of healthcare services among insured patients. This approach would discourage the unnecessary use of high-cost treatments, balancing service demand with cost containment. Additionally, implementing preventive care incentives could be instrumental in reducing ex-ante moral hazard. By encouraging patients to adopt healthier behaviors through reward programs or reduced insurance premiums, the need for reactive healthcare services would likely diminish.

Strengthening regulations on provider behavior is equally important in addressing healthcare inefficiencies. By establishing clear guidelines for healthcare providers and conducting regular audits, the overprescription of unnecessary procedures and treatments—driven by provider-induced demand—can be mitigated, ultimately curbing rising healthcare costs.

By implementing these reforms, Romania’s healthcare system could achieve a better balance between accessibility and sustainability, ensuring essential services remain available without encouraging the excessive use of medical resources. Given the significant impact of moral hazard on the system’s sustainability, it is imperative for policymakers to prioritize the introduction of co-payment systems and to strengthen provider accountability to ensure efficient use of public resources.

## Figures and Tables

**Table 1:** Summary of presented vignettes

Condition	Proposed Treatment	High-Cost Scenario	Low-Cost Scenario
Herniated Disc	Surgery/Physiotherapy	Surgery costing 20,000 RON	Delay surgery; intensive medical treatment and physiotherapy
Renal Cancer	Standard Treatment/New Medication	Chemotherapy with an 80% survival chance	Chemotherapy + new medication costing 15,000 RON/month
Hearing Loss	Traditional/Implantable Prosthesis	Implantable prosthesis costing 25,000–40,000 RON	Traditional hearing aid costing 10,000 RON

## Data Availability

The data presented in this study are available on request from the corresponding author.

## Supplementary Materials

The following supporting information can be downloaded at: https://www.mdpi.com/article/10.3390/healthcare12242519/s1.
